# Tri-*tert*-butyl­phospho­nium hy­droxy­tris­(penta­fluoro­phen­yl)borate

**DOI:** 10.1107/S1600536812018144

**Published:** 2012-04-28

**Authors:** Marcus Klahn, Anke Spannenberg, Uwe Rosenthal

**Affiliations:** a Leibniz-Institut für Katalyse e.V. an der Universität Rostock, Albert-Einstein-Strasse 29a, 18059 Rostock, Germany

## Abstract

The ionic title compound, C_12_H_28_P^+^·C_18_HBF_15_O^−^, was obtained by the stoichiometric reaction of ^*t*^Bu_3_P, B(C_6_F_5_)_3_ and water in toluene. A weak P—H⋯O hydrogen bond is observed in the crystal structure.

## Related literature

For general aspects of related compounds, see: Welch *et al.* (2007 ▶); Stephan & Erker (2010 ▶). For related structures, see: Roesler *et al.* (2003 ▶); Di Saverio *et al.* (2005 ▶); Welch & Stephan (2007 ▶).
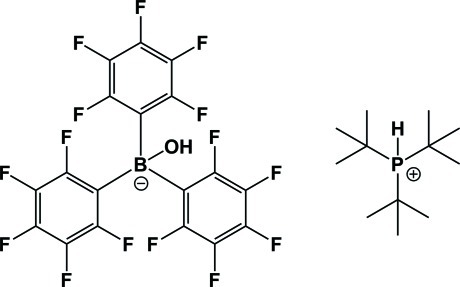


## Experimental

### 

#### Crystal data

C_12_H_28_P^+^·C_18_HBF_15_O^−^
*M* *_r_* = 732.31
Triclinic, 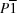

*a* = 9.798 (2) Å
*b* = 12.042 (2) Å
*c* = 15.389 (3) Å
α = 112.31 (3)°
β = 94.51 (3)°
γ = 108.93 (3)°
*V* = 1546.2 (8) Å^3^
*Z* = 2
Mo *K*α radiation
μ = 0.21 mm^−1^
*T* = 200 K
0.30 × 0.23 × 0.15 mm

#### Data collection

Stoe IPDS II diffractometer
24084 measured reflections
6557 independent reflections
4256 reflections with *I* > 2σ(*I*)
*R*_int_ = 0.031

#### Refinement

*R*[*F* ^2^ > 2σ(*F* ^2^)] = 0.030
*wR*(*F* ^2^) = 0.069
*S* = 0.84
6557 reflections
450 parameters
H atoms treated by a mixture of independent and constrained refinement
Δρ_max_ = 0.20 e Å^−3^
Δρ_min_ = −0.24 e Å^−3^

### 

Data collection: *X-AREA* (Stoe & Cie, 2005 ▶); cell refinement: *X-AREA*; data reduction: *X-RED32*; program(s) used to solve structure: *SHELXS97* (Sheldrick, 2008 ▶); program(s) used to refine structure: *SHELXL97* (Sheldrick, 2008 ▶); molecular graphics: *XP* in *SHELXTL* (Sheldrick, 2008 ▶); software used to prepare material for publication: *SHELXTL*.

## Supplementary Material

Crystal structure: contains datablock(s) I, global. DOI: 10.1107/S1600536812018144/hg5215sup1.cif

Structure factors: contains datablock(s) I. DOI: 10.1107/S1600536812018144/hg5215Isup2.hkl

Additional supplementary materials:  crystallographic information; 3D view; checkCIF report

## Figures and Tables

**Table 1:** Hydrogen-bond geometry (Å, °)

*D*—H⋯*A*	*D*—H	H⋯*A*	*D*⋯*A*	*D*—H⋯*A*
P1—H2⋯O1^i^	1.288 (14)	2.276 (14)	3.4080 (13)	144.6 (9)

Symmetry code: (i) 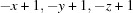.

**Fig. 1.**
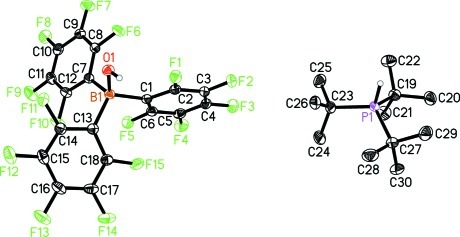
The molecular structure of the title compound with atom labels and 30% displacement ellipsoids. Hydrogen atoms at carbon atoms are omitted for clarity.

## supplementary crystallographic information

### Comment

The concept of "frustrated Lewis pairs" has been explored in depth and coined by Welch *et al.* (2007). The key issue is the sterical hindrance between the Lewis acids and bases preventing a classical adduct formation which gives rise to a unique reactivity due to the interaction of the basic and acidic centres. During the last years, this feature has been shown in reactions with a wide variety of reagents, these can be either small molecules like H_2_, CO_2_, and N_2_O, or larger ones like terminal olefins, alkynes, dienes, B–H bonds, disulfides and the C–O bonds of cyclic ethers (Stephan & Erker 2010). The reaction of tri-*tert-*butylphosphine, tris(pentafluorophenyl)borane and water proceeds in a similar way to the conversion of tris(pentafluorophenyl)borane with water in the presence of triethylamine (Di Saverio *et al.*, 2005). Roesler *et al.* (2003) obtained an intramolecular salt of the type 1-[N(H)Ph_2_]-2-[B(OH)(C_6_F_5_)_2_]C_6_H_4_ with a phenyl bridge between nitrogen and boron. The title compound consists of the phosphonium cation [tBu_3_PH]^+^ and the boranate anion [HOB(C_6_F_5_)_3_]^-^ (Figure 1), which are probably generated *via* subsequent protonation of the phosphine by the formed borane water adduct. The phosphonium cation is comparable to that in the compound [tBu_3_PH][HB(C_6_F_5_)_3_], which is the product of dihydrogen activation (Welch & Stephan 2007). Besides the unexceptional metric parameters both ions exhibit a geometry of a distorted tetrahedron at the phosphorous and the boron centre, respectively. Noteworthy, a weak P—H···O hydrogen bond was obtained with following geometric parameters: P1—H2 1.288 (14), H2···O1 2.276 (14), P1···O1 3.4080 (13) Å, P1—H2···O1 144.6 (9)°.

### Experimental

Solid tris(pentafluorophenyl)borane (0.256 g, 0.5 mmol) and tri-*tert*-butylphosphine (0.101 g, 0.5 mmol) were dissolved in 20 ml of toluene resulting in a pale yellow solution. After stirring this mixture for 30 minutes 9 µ*L* of water (0.5 mmol) was added. The reaction mixture was allowed to stir for 12 h at 40 °C during which the solution turned colorless. The reaction was concentrated until the first precipitate was formed, which was resolved by gentle heating. Leaving the solution at -78 °C gave colorless prisms in an isolated yield of 0.127 g (35%) The colorless compound was fully characterized by standard analytical methods, NMR (295 K, CDCl_3_): ^1^H: d = 5.96 (d, 1H, ^1^*J*_HP_ = 451 Hz, P*H*), 2.05 (br s, 1H, *H*OB), 1.58 (d, 27H, ^3^*J*_HP_ = 15.3 Hz, {(C*H*_3_)_3_C}_3_). ^11^B: d = -3.74 (*s*). ^31^P{^1^H}: d = 54.8 (*s*). ^31^P: d = 54.8 (dm, ^1^*J*_PH_ = 452 Hz, ^3^*J*_HP_ = 15.4 Hz). ^19^F: d = -135.0 (d, 6 F, ^3^*J*_FF_ = 21.6 Hz, *o*-F), -161.8 (t, 3 F, ^3^*J*_FF_ = 20.1 Hz, *p*-F), -165.5 (m, 6 F, *m*-F).

### Refinement

H1 and H2 were found in a difference Fourier map and refined isotropically. All other H atoms were placed in idealized positions with d(C—H) = 0.98 Å and refined using a riding model with *U*_iso_(H) fixed at 1.5 *U*_eq_(C).

### Crystal data

**Table:**

C_12_H_28_P^+^·C_18_HBF_15_O^−^	*Z* = 2
*M**_r_* = 732.31	*F*(000) = 744
Triclinic, *P*1	*D*_x_ = 1.573 Mg m^−^^3^
*a* = 9.798 (2) Å	Mo *K*α radiation, λ = 0.71073 Å
*b* = 12.042 (2) Å	Cell parameters from 5729 reflections
*c* = 15.389 (3) Å	θ = 1.5–27.2°
α = 112.31 (3)°	µ = 0.21 mm^−^^1^
β = 94.51 (3)°	*T* = 200 K
γ = 108.93 (3)°	Prism, colourless
*V* = 1546.2 (8) Å^3^	0.30 × 0.23 × 0.15 mm

### Data collection

**Table:**

Stoe IPDS II diffractometer	4256 reflections with *I* > 2σ(*I*)
Radiation source: fine-focus sealed tube	*R*_int_ = 0.031
Graphite monochromator	θ_max_ = 26.7°, θ_min_ = 1.5°
ω scans	*h* = −12→12
24084 measured reflections	*k* = −15→15
6557 independent reflections	*l* = −19→19

### Refinement

**Table:**

Refinement on *F*^2^	Primary atom site location: structure-invariant direct methods
Least-squares matrix: full	Secondary atom site location: difference Fourier map
*R*[*F*^2^ > 2σ(*F*^2^)] = 0.030	Hydrogen site location: inferred from neighbouring sites
*wR*(*F*^2^) = 0.069	H atoms treated by a mixture of independent and constrained refinement
*S* = 0.84	*w* = 1/[σ^2^(*F*_o_^2^) + (0.0383*P*)^2^] where *P* = (*F*_o_^2^ + 2*F*_c_^2^)/3
6557 reflections	(Δ/σ)_max_ = 0.001
450 parameters	Δρ_max_ = 0.20 e Å^−^^3^
0 restraints	Δρ_min_ = −0.24 e Å^−^^3^

### Special details

**Table:**

Geometry. All e.s.d.'s (except the e.s.d. in the dihedral angle between two l.s. planes) are estimated using the full covariance matrix. The cell e.s.d.'s are taken into account individually in the estimation of e.s.d.'s in distances, angles and torsion angles; correlations between e.s.d.'s in cell parameters are only used when they are defined by crystal symmetry. An approximate (isotropic) treatment of cell e.s.d.'s is used for estimating e.s.d.'s involving l.s. planes.
Refinement. Refinement of *F*^2^ against ALL reflections. The weighted *R*-factor *wR* and goodness of fit *S* are based on *F*^2^, conventional *R*-factors *R* are based on *F*, with *F* set to zero for negative *F*^2^. The threshold expression of *F*^2^ > σ(*F*^2^) is used only for calculating *R*-factors(gt) *etc*. and is not relevant to the choice of reflections for refinement. *R*-factors based on *F*^2^ are statistically about twice as large as those based on *F*, and *R*- factors based on ALL data will be even larger.

### Fractional atomic coordinates and isotropic or equivalent isotropic displacement parameters (Å^2^)

**Table:**

	*x*	*y*	*z*	*U*_iso_*/*U*_eq_	
C1	0.23991 (17)	0.30327 (14)	0.67250 (11)	0.0311 (3)	
C2	0.33984 (17)	0.36491 (15)	0.63024 (11)	0.0341 (3)	
C3	0.37910 (19)	0.30153 (17)	0.54731 (12)	0.0391 (4)	
C4	0.3198 (2)	0.16833 (17)	0.50170 (11)	0.0421 (4)	
C5	0.22222 (19)	0.10115 (15)	0.54084 (12)	0.0404 (4)	
C6	0.18568 (18)	0.16856 (15)	0.62327 (11)	0.0348 (4)	
C7	−0.00162 (17)	0.30725 (14)	0.73956 (10)	0.0311 (3)	
C8	−0.08453 (17)	0.32198 (14)	0.67075 (11)	0.0327 (3)	
C9	−0.23652 (18)	0.26740 (15)	0.64192 (11)	0.0366 (4)	
C10	−0.31631 (18)	0.19121 (16)	0.68202 (12)	0.0390 (4)	
C11	−0.24162 (19)	0.17148 (16)	0.74982 (12)	0.0397 (4)	
C12	−0.08921 (18)	0.22759 (15)	0.77625 (11)	0.0362 (4)	
C13	0.25420 (17)	0.37488 (14)	0.86700 (11)	0.0320 (3)	
C14	0.22187 (18)	0.44094 (15)	0.95346 (11)	0.0365 (4)	
C15	0.2796 (2)	0.45011 (17)	1.04117 (12)	0.0443 (4)	
C16	0.37549 (19)	0.39040 (18)	1.04639 (12)	0.0453 (4)	
C17	0.41270 (18)	0.32459 (16)	0.96440 (13)	0.0420 (4)	
C18	0.35269 (18)	0.31783 (15)	0.87707 (11)	0.0361 (4)	
C19	0.8368 (2)	0.14093 (16)	0.09712 (12)	0.0437 (4)	
C20	0.9725 (2)	0.1684 (2)	0.05404 (14)	0.0576 (5)	
H20A	1.0453	0.1429	0.0805	0.086*	
H20B	1.0168	0.2614	0.0703	0.086*	
H20C	0.9424	0.1185	−0.0163	0.086*	
C21	0.7709 (3)	−0.00456 (17)	0.07480 (16)	0.0636 (6)	
H21A	0.7464	−0.0570	0.0048	0.095*	
H21B	0.6809	−0.0233	0.0994	0.095*	
H21C	0.8436	−0.0258	0.1060	0.095*	
C22	0.7245 (2)	0.1750 (2)	0.04734 (13)	0.0587 (5)	
H22A	0.7639	0.2688	0.0660	0.088*	
H22B	0.6308	0.1499	0.0672	0.088*	
H22C	0.7072	0.1280	−0.0228	0.088*	
C23	0.7343 (2)	0.20913 (17)	0.29229 (14)	0.0449 (4)	
C24	0.7288 (3)	0.0981 (2)	0.32003 (18)	0.0687 (6)	
H24A	0.6500	0.0829	0.3546	0.103*	
H24B	0.8243	0.1218	0.3617	0.103*	
H24C	0.7087	0.0187	0.2615	0.103*	
C25	0.5834 (2)	0.1725 (2)	0.22956 (17)	0.0599 (5)	
H25A	0.5591	0.0906	0.1724	0.090*	
H25B	0.5872	0.2413	0.2094	0.090*	
H25C	0.5072	0.1622	0.2667	0.090*	
C26	0.7618 (2)	0.33412 (19)	0.38274 (14)	0.0550 (5)	
H26A	0.6741	0.3231	0.4102	0.082*	
H26B	0.7811	0.4070	0.3651	0.082*	
H26C	0.8476	0.3519	0.4305	0.082*	
C27	1.0711 (2)	0.27172 (17)	0.29329 (13)	0.0446 (4)	
C28	1.0933 (3)	0.3432 (2)	0.40278 (14)	0.0666 (6)	
H28A	1.1960	0.3669	0.4344	0.100*	
H28B	1.0257	0.2863	0.4258	0.100*	
H28C	1.0726	0.4220	0.4182	0.100*	
C29	1.1911 (2)	0.3614 (2)	0.26475 (16)	0.0611 (5)	
H29A	1.1724	0.4403	0.2759	0.092*	
H29B	1.1889	0.3162	0.1963	0.092*	
H29C	1.2887	0.3850	0.3038	0.092*	
C30	1.0909 (2)	0.1425 (2)	0.26836 (17)	0.0614 (5)	
H30A	1.0789	0.0978	0.1985	0.092*	
H30B	1.0161	0.0876	0.2892	0.092*	
H30C	1.1901	0.1597	0.3015	0.092*	
F1	0.40912 (11)	0.49630 (8)	0.67088 (7)	0.0447 (2)	
F2	0.47413 (12)	0.36859 (11)	0.50949 (8)	0.0551 (3)	
F3	0.35817 (14)	0.10532 (11)	0.42120 (7)	0.0622 (3)	
F4	0.16501 (13)	−0.02981 (9)	0.49855 (7)	0.0580 (3)	
F5	0.09291 (11)	0.09601 (8)	0.65966 (7)	0.0454 (2)	
F6	−0.01457 (10)	0.39236 (9)	0.62464 (7)	0.0459 (2)	
F7	−0.30771 (11)	0.28541 (11)	0.57324 (8)	0.0575 (3)	
F8	−0.46452 (11)	0.13439 (11)	0.65281 (8)	0.0570 (3)	
F9	−0.31768 (12)	0.09451 (11)	0.78874 (8)	0.0612 (3)	
F10	−0.02586 (11)	0.19765 (10)	0.84141 (7)	0.0538 (3)	
F11	0.12531 (11)	0.49906 (10)	0.95367 (7)	0.0494 (3)	
F12	0.24301 (13)	0.51617 (12)	1.12182 (7)	0.0662 (3)	
F13	0.43409 (12)	0.39742 (12)	1.13126 (8)	0.0681 (3)	
F14	0.51001 (12)	0.26803 (10)	0.96855 (8)	0.0599 (3)	
F15	0.39801 (12)	0.25147 (10)	0.80060 (7)	0.0517 (3)	
B1	0.1813 (2)	0.37833 (16)	0.76764 (12)	0.0312 (4)	
O1	0.21571 (15)	0.51498 (10)	0.78731 (8)	0.0353 (3)	
P1	0.88590 (5)	0.24737 (4)	0.22942 (3)	0.03315 (10)	
H1	0.304 (3)	0.549 (2)	0.7996 (15)	0.067 (8)*	
H2	0.9035 (16)	0.3605 (13)	0.2343 (10)	0.027 (4)*	

### Atomic displacement parameters (Å^2^)

**Table:**

	*U*^11^	*U*^22^	*U*^33^	*U*^12^	*U*^13^	*U*^23^
C1	0.0295 (8)	0.0347 (8)	0.0334 (8)	0.0150 (7)	0.0059 (7)	0.0172 (7)
C2	0.0318 (9)	0.0358 (8)	0.0390 (8)	0.0150 (7)	0.0073 (7)	0.0189 (7)
C3	0.0397 (10)	0.0534 (10)	0.0409 (9)	0.0263 (8)	0.0163 (8)	0.0289 (8)
C4	0.0503 (11)	0.0557 (11)	0.0319 (8)	0.0342 (9)	0.0142 (8)	0.0182 (8)
C5	0.0458 (10)	0.0352 (9)	0.0377 (9)	0.0213 (8)	0.0025 (8)	0.0097 (7)
C6	0.0345 (9)	0.0348 (8)	0.0398 (9)	0.0145 (7)	0.0082 (7)	0.0198 (7)
C7	0.0332 (9)	0.0307 (8)	0.0319 (8)	0.0134 (7)	0.0091 (7)	0.0148 (7)
C8	0.0334 (9)	0.0343 (8)	0.0352 (8)	0.0134 (7)	0.0114 (7)	0.0187 (7)
C9	0.0335 (9)	0.0427 (9)	0.0374 (9)	0.0178 (8)	0.0062 (7)	0.0187 (7)
C10	0.0257 (9)	0.0424 (9)	0.0425 (9)	0.0095 (7)	0.0073 (7)	0.0146 (8)
C11	0.0378 (10)	0.0387 (9)	0.0397 (9)	0.0057 (8)	0.0136 (8)	0.0203 (7)
C12	0.0370 (9)	0.0382 (8)	0.0350 (8)	0.0116 (7)	0.0050 (7)	0.0204 (7)
C13	0.0283 (8)	0.0294 (8)	0.0370 (8)	0.0079 (7)	0.0060 (7)	0.0159 (7)
C14	0.0313 (9)	0.0385 (9)	0.0396 (9)	0.0100 (7)	0.0082 (7)	0.0194 (7)
C15	0.0406 (10)	0.0492 (10)	0.0348 (9)	0.0055 (8)	0.0103 (8)	0.0192 (8)
C16	0.0354 (10)	0.0546 (10)	0.0408 (10)	0.0007 (8)	−0.0006 (8)	0.0310 (9)
C17	0.0294 (9)	0.0408 (9)	0.0577 (11)	0.0063 (8)	−0.0014 (8)	0.0312 (9)
C18	0.0350 (9)	0.0321 (8)	0.0403 (9)	0.0117 (7)	0.0049 (7)	0.0164 (7)
C19	0.0522 (11)	0.0369 (9)	0.0435 (9)	0.0211 (8)	0.0118 (8)	0.0153 (8)
C20	0.0748 (15)	0.0619 (12)	0.0485 (11)	0.0353 (11)	0.0290 (10)	0.0258 (10)
C21	0.0714 (15)	0.0362 (10)	0.0717 (14)	0.0204 (10)	0.0136 (11)	0.0124 (10)
C22	0.0717 (14)	0.0570 (12)	0.0429 (10)	0.0320 (11)	0.0009 (10)	0.0133 (9)
C23	0.0444 (10)	0.0458 (10)	0.0623 (11)	0.0212 (8)	0.0261 (9)	0.0354 (9)
C24	0.0778 (16)	0.0680 (13)	0.1029 (17)	0.0370 (12)	0.0521 (14)	0.0663 (13)
C25	0.0424 (12)	0.0564 (12)	0.0889 (15)	0.0177 (10)	0.0245 (11)	0.0386 (11)
C26	0.0655 (13)	0.0676 (12)	0.0562 (11)	0.0395 (11)	0.0346 (10)	0.0355 (10)
C27	0.0419 (10)	0.0530 (10)	0.0504 (10)	0.0231 (9)	0.0111 (8)	0.0296 (9)
C28	0.0649 (14)	0.0878 (16)	0.0520 (12)	0.0383 (13)	0.0014 (10)	0.0298 (11)
C29	0.0392 (11)	0.0630 (13)	0.0807 (14)	0.0134 (10)	0.0118 (10)	0.0362 (12)
C30	0.0594 (13)	0.0714 (13)	0.0835 (15)	0.0420 (11)	0.0229 (11)	0.0483 (12)
F1	0.0430 (6)	0.0367 (5)	0.0561 (6)	0.0122 (4)	0.0195 (5)	0.0226 (5)
F2	0.0597 (7)	0.0738 (7)	0.0633 (6)	0.0378 (6)	0.0376 (6)	0.0465 (6)
F3	0.0838 (8)	0.0728 (7)	0.0429 (6)	0.0491 (7)	0.0274 (6)	0.0195 (5)
F4	0.0728 (8)	0.0373 (5)	0.0535 (6)	0.0250 (5)	0.0081 (5)	0.0069 (5)
F5	0.0507 (6)	0.0336 (5)	0.0567 (6)	0.0164 (5)	0.0192 (5)	0.0227 (5)
F6	0.0402 (6)	0.0579 (6)	0.0502 (6)	0.0138 (5)	0.0100 (4)	0.0383 (5)
F7	0.0407 (6)	0.0777 (7)	0.0643 (7)	0.0211 (6)	0.0008 (5)	0.0443 (6)
F8	0.0284 (6)	0.0688 (7)	0.0674 (7)	0.0095 (5)	0.0086 (5)	0.0309 (6)
F9	0.0486 (7)	0.0672 (7)	0.0612 (7)	−0.0018 (5)	0.0127 (5)	0.0409 (6)
F10	0.0472 (6)	0.0596 (6)	0.0576 (6)	0.0051 (5)	−0.0003 (5)	0.0433 (5)
F11	0.0513 (6)	0.0613 (6)	0.0453 (5)	0.0316 (5)	0.0204 (5)	0.0227 (5)
F12	0.0705 (8)	0.0830 (8)	0.0378 (6)	0.0220 (7)	0.0194 (5)	0.0233 (6)
F13	0.0519 (7)	0.1004 (9)	0.0526 (6)	0.0096 (6)	−0.0018 (5)	0.0525 (7)
F14	0.0465 (6)	0.0617 (7)	0.0822 (8)	0.0207 (5)	−0.0019 (6)	0.0451 (6)
F15	0.0572 (7)	0.0556 (6)	0.0500 (6)	0.0372 (5)	0.0086 (5)	0.0184 (5)
B1	0.0332 (10)	0.0296 (9)	0.0341 (9)	0.0124 (8)	0.0079 (8)	0.0168 (7)
O1	0.0356 (7)	0.0298 (6)	0.0430 (6)	0.0130 (5)	0.0090 (5)	0.0179 (5)
P1	0.0358 (2)	0.0319 (2)	0.0392 (2)	0.01505 (18)	0.01272 (18)	0.02023 (18)

### Geometric parameters (Å, º)

**Table:**

C1—C2	1.388 (2)	C19—P1	1.8667 (19)
C1—C6	1.390 (2)	C20—H20A	0.9800
C1—B1	1.660 (2)	C20—H20B	0.9800
C2—F1	1.3567 (18)	C20—H20C	0.9800
C2—C3	1.374 (2)	C21—H21A	0.9800
C3—F2	1.3461 (19)	C21—H21B	0.9800
C3—C4	1.371 (2)	C21—H21C	0.9800
C4—F3	1.3405 (19)	C22—H22A	0.9800
C4—C5	1.375 (2)	C22—H22B	0.9800
C5—F4	1.3471 (19)	C22—H22C	0.9800
C5—C6	1.369 (2)	C23—C25	1.531 (3)
C6—F5	1.3562 (18)	C23—C26	1.537 (3)
C7—C8	1.383 (2)	C23—C24	1.538 (2)
C7—C12	1.389 (2)	C23—P1	1.8639 (18)
C7—B1	1.653 (2)	C24—H24A	0.9800
C8—F6	1.3593 (17)	C24—H24B	0.9800
C8—C9	1.373 (2)	C24—H24C	0.9800
C9—F7	1.3442 (18)	C25—H25A	0.9800
C9—C10	1.369 (2)	C25—H25B	0.9800
C10—F8	1.3410 (19)	C25—H25C	0.9800
C10—C11	1.364 (2)	C26—H26A	0.9800
C11—F9	1.3478 (18)	C26—H26B	0.9800
C11—C12	1.376 (2)	C26—H26C	0.9800
C12—F10	1.3574 (17)	C27—C28	1.532 (3)
C13—C18	1.384 (2)	C27—C30	1.536 (2)
C13—C14	1.389 (2)	C27—C29	1.536 (3)
C13—B1	1.657 (2)	C27—P1	1.8710 (19)
C14—F11	1.3466 (19)	C28—H28A	0.9800
C14—C15	1.371 (2)	C28—H28B	0.9800
C15—F12	1.345 (2)	C28—H28C	0.9800
C15—C16	1.369 (3)	C29—H29A	0.9800
C16—F13	1.3451 (18)	C29—H29B	0.9800
C16—C17	1.362 (3)	C29—H29C	0.9800
C17—F14	1.3497 (19)	C30—H30A	0.9800
C17—C18	1.386 (2)	C30—H30B	0.9800
C18—F15	1.3489 (19)	C30—H30C	0.9800
C19—C20	1.528 (3)	B1—O1	1.469 (2)
C19—C22	1.538 (2)	O1—H1	0.79 (2)
C19—C21	1.542 (2)	P1—H2	1.288 (14)
			
C2—C1—C6	112.68 (14)	H21A—C21—H21C	109.5
C2—C1—B1	125.07 (13)	H21B—C21—H21C	109.5
C6—C1—B1	122.16 (14)	C19—C22—H22A	109.5
F1—C2—C3	114.64 (14)	C19—C22—H22B	109.5
F1—C2—C1	120.90 (14)	H22A—C22—H22B	109.5
C3—C2—C1	124.45 (15)	C19—C22—H22C	109.5
F2—C3—C4	119.39 (15)	H22A—C22—H22C	109.5
F2—C3—C2	120.84 (15)	H22B—C22—H22C	109.5
C4—C3—C2	119.77 (15)	C25—C23—C26	106.75 (16)
F3—C4—C3	120.34 (16)	C25—C23—C24	108.96 (16)
F3—C4—C5	120.90 (16)	C26—C23—C24	110.57 (16)
C3—C4—C5	118.75 (15)	C25—C23—P1	111.06 (13)
F4—C5—C6	120.76 (16)	C26—C23—P1	107.30 (13)
F4—C5—C4	119.86 (15)	C24—C23—P1	112.07 (13)
C6—C5—C4	119.38 (15)	C23—C24—H24A	109.5
F5—C6—C5	116.17 (14)	C23—C24—H24B	109.5
F5—C6—C1	118.86 (14)	H24A—C24—H24B	109.5
C5—C6—C1	124.94 (15)	C23—C24—H24C	109.5
C8—C7—C12	112.44 (14)	H24A—C24—H24C	109.5
C8—C7—B1	120.11 (13)	H24B—C24—H24C	109.5
C12—C7—B1	127.43 (13)	C23—C25—H25A	109.5
F6—C8—C9	115.40 (13)	C23—C25—H25B	109.5
F6—C8—C7	119.61 (14)	H25A—C25—H25B	109.5
C9—C8—C7	124.97 (14)	C23—C25—H25C	109.5
F7—C9—C10	119.58 (15)	H25A—C25—H25C	109.5
F7—C9—C8	120.82 (14)	H25B—C25—H25C	109.5
C10—C9—C8	119.58 (14)	C23—C26—H26A	109.5
F8—C10—C11	121.04 (15)	C23—C26—H26B	109.5
F8—C10—C9	120.31 (15)	H26A—C26—H26B	109.5
C11—C10—C9	118.63 (15)	C23—C26—H26C	109.5
F9—C11—C10	119.66 (15)	H26A—C26—H26C	109.5
F9—C11—C12	120.45 (15)	H26B—C26—H26C	109.5
C10—C11—C12	119.86 (14)	C28—C27—C30	109.52 (16)
F10—C12—C11	115.05 (14)	C28—C27—C29	106.15 (17)
F10—C12—C7	120.43 (14)	C30—C27—C29	110.30 (16)
C11—C12—C7	124.51 (14)	C28—C27—P1	110.65 (14)
C18—C13—C14	113.37 (14)	C30—C27—P1	112.04 (14)
C18—C13—B1	127.75 (14)	C29—C27—P1	108.01 (12)
C14—C13—B1	118.80 (13)	C27—C28—H28A	109.5
F11—C14—C15	115.91 (15)	C27—C28—H28B	109.5
F11—C14—C13	119.51 (14)	H28A—C28—H28B	109.5
C15—C14—C13	124.57 (16)	C27—C28—H28C	109.5
F12—C15—C16	119.63 (15)	H28A—C28—H28C	109.5
F12—C15—C14	121.01 (17)	H28B—C28—H28C	109.5
C16—C15—C14	119.36 (17)	C27—C29—H29A	109.5
F13—C16—C17	119.98 (17)	C27—C29—H29B	109.5
F13—C16—C15	120.87 (18)	H29A—C29—H29B	109.5
C17—C16—C15	119.15 (15)	C27—C29—H29C	109.5
F14—C17—C16	119.98 (15)	H29A—C29—H29C	109.5
F14—C17—C18	120.08 (17)	H29B—C29—H29C	109.5
C16—C17—C18	119.93 (15)	C27—C30—H30A	109.5
F15—C18—C13	121.25 (14)	C27—C30—H30B	109.5
F15—C18—C17	115.12 (14)	H30A—C30—H30B	109.5
C13—C18—C17	123.63 (16)	C27—C30—H30C	109.5
C20—C19—C22	106.21 (15)	H30A—C30—H30C	109.5
C20—C19—C21	109.34 (16)	H30B—C30—H30C	109.5
C22—C19—C21	109.95 (16)	O1—B1—C7	106.05 (13)
C20—C19—P1	110.77 (13)	O1—B1—C13	107.91 (13)
C22—C19—P1	108.44 (12)	C7—B1—C13	111.45 (13)
C21—C19—P1	111.96 (13)	O1—B1—C1	111.68 (13)
C19—C20—H20A	109.5	C7—B1—C1	106.95 (13)
C19—C20—H20B	109.5	C13—B1—C1	112.63 (12)
H20A—C20—H20B	109.5	B1—O1—H1	106.0 (16)
C19—C20—H20C	109.5	C23—P1—C19	114.47 (9)
H20A—C20—H20C	109.5	C23—P1—C27	114.13 (8)
H20B—C20—H20C	109.5	C19—P1—C27	113.94 (9)
C19—C21—H21A	109.5	C23—P1—H2	105.0 (6)
C19—C21—H21B	109.5	C19—P1—H2	103.9 (6)
H21A—C21—H21B	109.5	C27—P1—H2	103.7 (6)
C19—C21—H21C	109.5		

### Hydrogen-bond geometry (Å, º)

**Table:**

*D*—H···*A*	*D*—H	H···*A*	*D*···*A*	*D*—H···*A*
P1—H2···O1^i^	1.288 (14)	2.276 (14)	3.4080 (13)	144.6 (9)

Symmetry code: (i) −*x*+1, −*y*+1, −*z*+1.
